# Influence of tobacco, alcohol consumption, eating habits and physical activity in nursing students*

**DOI:** 10.1590/1518-8345.3198.3230

**Published:** 2020-02-03

**Authors:** Pedro Manuel Rodríguez-Muñoz, Juan Manuel Carmona-Torres, María Aurora Rodríguez-Borrego

**Affiliations:** 1 Universidad de Córdoba, Córdoba, Spain.; 2 Instituto Maimónides de Investigación Biomédica de Córdoba, Córdoba, Spain.; 3 Universidad Pontificia de Salamanca, Salamanca, Spain.; 4 Universidad de Castilla-La Mancha, Toledo, Spain.

**Keywords:** Alcohol, Tobacco, Eating Habits, Physical Activity, University Students, Nursing, Álcool, Tabaco, Hábitos Alimentares, Atividade Física, Estudantes Universitários, Enfermagem, Alcohol, Tabaco, Hábitos Alimenticios, Actividad Física, Estudiantes Universitarios, Enfermería

## Abstract

**Objective:**

to determine the consumption of alcohol, tobacco, eating habits, and physical activity among nursing students and to detect whether being a nursing student is a protective factor against these habits.

**Method:**

a questionnaire was used to collect information on age, academic year, sex, alcohol and tobacco consumption, physical activity, and healthy eating. An information sheet and informed consent form were given. The sample was composed of 264 students aged between 18-30 years from four academic years.

**Results:**

of the total sample, 15.5% smoked, 83.7% consumed alcoholic beverages and 97.2% consumed over the weekend. The total of 68.6% did not practice exercises and 70.5% needed changes in their diet.

**Conclusion:**

nursing students have high levels of alcohol consumption and low levels of smoking compared with other studies. The higher the academic year, the lower the age of onset of tobacco consumption. The number of men who exercised was higher, which is considered a protective factor against alcohol and tobacco consumption and is related to a healthy diet. Smoking has a negative influence on diet. The students needed to change their diet. Finally, being a nursing student is not considered a protective factor against alcohol and tobacco consumption, nor having good eating habits and exercising.

## Abstract (pt)

**Objetivo:**

determinar o consumo de álcool e tabaco, os hábitos alimentares e a atividade física entre estudantes de enfermagem e verificar se o fato de ser estudante de enfermagem é fator de proteção contra estes hábitos.

**Método:**

utilizou-se questionário para coletar informações sobre idade, ano letivo, sexo, consumo de álcool e tabaco, atividade física e alimentação saudável. Foram entregues uma folha com informações e um termo de consentimento livre e esclarecido. A amostra foi composta por 264 estudantes com idades entre 18 e 30 anos, de quatro anos acadêmicos.

**Resultados:**

do total da amostra, 15,5% fumavam, 83,7% consumiam bebidas alcoólicas e 97,2% consumiam no final de semana. Um total de 68,6% não praticava exercícios, e 70,5% necessitavam de alterações na dieta.

**Conclusão:**

estudantes de enfermagem apresentam altos níveis de consumo de álcool e baixos níveis de tabagismo em comparação com outros estudos. Quanto maior é o ano letivo, menor é a idade em que iniciaram o consumo de tabaco. O número de homens que se exercitavam foi maior, o que é considerado fator de proteção contra o consumo de álcool e tabaco e está relacionado a uma dieta saudável. Fumar tem influência negativa na dieta. Os estudantes precisavam mudar sua dieta. Por fim, ser estudante de enfermagem não é considerado fator de proteção contra o consumo de álcool e tabaco, nem significa ter bons hábitos alimentares e praticar exercícios.

## Abstract (es)

**Objetivo:**

determinar el consumo de alcohol, tabaco, hábitos alimenticios y actividad física entre los estudiantes de enfermería y detectar si ser estudiante de enfermería es un factor protector contra estos hábitos.

**Método:**

se utilizó un cuestionario para recopilar información sobre la edad, el año académico, el sexo, el consumo de alcohol y tabaco, la actividad física y la alimentación saludable. Se presentó una hoja de información y un formulario de consentimiento informado. La muestra estaba compuesta por 264 estudiantes que tenían entre 18 y 30 años, a partir de cuatro años académicos.

**Resultados:**

del total de la muestra, el 15,5% fumaba, el 83,7% consumía bebidas alcohólicas y el 97,2% consumía alcohol durante el fin de semana. El total del 68,6% no practica ejercicio físico y el 70,5% necesita cambios en su dieta.

**Conclusión:**

los estudiantes de enfermería tienen altos niveles de consumo de alcohol y bajos niveles de tabaquismo en comparación con otros estudios. A mayor curso académico, menor será la edad de inicio del consumo de tabaco. El número de hombres que practican actividad física fue mayor, lo que es considerado un factor protector contra el consumo de alcohol y tabaco y está relacionado con una dieta saludable. Fumar influye negativamente en la dieta. Los estudiantes necesitaban cambiar sus dietas. Por último, ser estudiante de enfermería no es considerado un factor protector contra el consumo de alcohol y tabaco, ni para que se tenga buenos hábitos alimenticios ni para que se practique actividad física.

## Introduction

Drugs are psychoactive substances that affects perception, mood, consciousness, and behavior^(^ 1 ^)^. Drugs with the highest prevalence of consumption in the general population are consumed at high levels by young people aged between 14 and 18 years. As of 2013, the most consumed drugs were alcohol and tobacco (78% and 41%, respectively), followed by cannabis (9%)^(^ 2 ^)^. In Spain, alcoholic beverages are the most consumed psychoactive substances among people aged between 15 and 64 years^(^ 2 ^)^. In addition, alcohol consumption has an important relationship with mortality and disease in Spain^(^ 3 ^)^. 

In general, health science students are more exposed to information about the risk factors associated with health^(^ 4 ^)^. Most nursing students are young and will become future health professionals, and several factors relate both protection and risk in terms of alcohol consumption^(^ 5 ^)^. The Spanish Observatory on Drugs and Drug Addictions^(^ 2 ^)^ conducted a Survey on Drugs and Alcohol in Spain (EDADES). According to the results, of the people aged between 15 and 64 years, 73% had ever consumed tobacco, 41% had used it in the last year, 38% had used it in the last month, and 31% use it daily. 

University students have a high rate of tobacco consumption, which often begins in adolescence and is strengthened at university^(^ 6 ^)^. Also, they are a vulnerable group regarding nutrition. The college period is when most students assume responsibility for their diets for the first time^(^ 7 ^)^. There is an association between nutrition and the consumption of psychoactive substances. Alcohol, for instance, inhibits food intake, while cannabis stimulates it. The consumption of psychoactive substances generates calcium deficiency^(^ 8 ^)^. The impact of alcohol consumption on the nutritional status can turn into primary and secondary malnutrition. Furthermore, alcohol interferes with the absorption of nutrients in the small intestine^(^ 8 ^)^.

According to the World Health Organization^(^ 9 ^)^, physical inactivity is the fourth leading risk factor for mortality in the world. Exercise is related to behaviors that enhance health and to a more positive self-perception^(^ 10 ^)^. Its absence is related to the consumption of tobacco, alcohol, and other drugs.

We believe no recent studies on nursing students address their academic training acts as a protective factor against tobacco and alcohol consumption, or having good eating habits and exercising. For this reason, it is worthy investigating if health knowledge affects these different habits.

The objective is to determine alcohol and tobacco consumption, eating habits, and physical activity among nursing students, as well as whether being a nursing student is a protective factor against tobacco and alcohol consumption; or having good eating habits and exercising.

## Method

This cross-sectional study asked 504 nursing students from the University of Córdoba (Spain) to participate. Of these, 305 students accepted the invitation. The data collection period was October 2016. The age range of the participants was 18 to 30 years.

Approval for this study was obtained from the Faculty of Medicine and Nursing and from the Research Ethics Committee of the province of Córdoba (Act no. 257, ref. 3291). We also went to the classrooms of each undergraduate course and obtained permission from the students’ professors. The students present in class were invited to participate in the study and informed of the procedure. Any possible doubts were also addressed, and an information sheet and informed consent form were handed out. 

A sociodemographic questionnaire was used to collect personal data about sex, age, and academic year. A tobacco, alcohol, and physical activity questionnaire^(^ 11 ^)^ was also used. The self-administered questionnaire is anonymous and has direct questions. It consists of three scales: tobacco consumption, alcohol consumption, and physical activity. The variables were frequency, hour of the first cigarette, age of onset, tobacco and alcohol consumption background of family or friends, anti-smoking campaigns, social pressure, type of alcoholic beverage, type of physical activity and passage through the university. This instrument is a useful tool to assess life habits of university students^(^ 11 ^)^.

The Spanish diet quality according to the healthy eating index (IASE) was used to determine the diet quality of the study population^(^ 12 ^)^. It is based on the methodology of the North American Healthy Eating Index (HEI)^(^ 13 ^)^. It consists of 10 variables that represent the food groups consumed daily, weekly, and occasionally, as well as the variety of the diet according to the recommendations proposed by the Spanish Society of Community Nutrition (SENC)^(^ 12 ^)^. The sum of the score of each variable can be used to classify a person’s diet into three categories: “healthy” if the score is higher than 80, “needs changes” if the score is lower than or equal to 80 and higher than 50, and “inadequate” if it is lower than or equal to 50^(^ 12 ^,^ 14 ^)^. This questionnaire was applied in Spanish^(^ 12 ^)^ and to university populations^(^ 14 ^)^.

The results were analyzed using Statistical Package for the Social Sciences (SPSS) v.23, a program for statistical analysis. The qualitative variables are described as counts (n) and percentages (%), and quantitative variables are summarized as the mean (m) and standard error of the mean (SD). Different variables are related using the χ² test in the case of qualitative variables and an analysis of variance for quantitative data. All contrasts were bilateral, and in all statistical tests, values were considered significant if their confidence level was 95% (*p*=0.05).

## Results

At first, 305 students agreed to participate in the study, but 28 were not within the age range and 13 did not fill out the whole questionnaire (they only filled out sociodemographic questions). Therefore, the final sample was composed of 264 students. The mean age (SD) was 20.79 years. There were 52 participants in their freshman year, 58 were sophomore, 59 were in their junior year, and 95 were seniors. The sample was 16.7% men and 83.3% women.

Regarding tobacco consumption, 84.5% of the students did not smoke, of these, 67% had never smoked. Furthermore, 7.2% smoked daily. Of the 15.5% who smoked, 12.5% smoke fewer than 10 cigarettes per day. As to sex, 20.5% of men and 14.6% of women smoked. A total of 4.9% started smoking for the first time between the ages of 13 and 15, 11.7% started between 16 and 18 years, and 7.2% started at 18. No student smoked for the first time before the age of 13. Significant differences were observed in relation to the academic year: the higher the year, the lower the age of onset of smoking. Among those in their senior year, 10.5% (*p*=0.037) started smoking between the ages of 13 and 15, while among those in their freshman year, nobody started before the age of 16. After completing university education, 2.7% of nursing students at the University of Córdoba claim to have quit smoking, 6.1% smoke less, 6.4% have not changed, and 4.2% smoke more.

Regarding the environment, 72.3% spent most of the day with someone who smokes sometimes. Significant differences were found among sophomores, in which 48.3% (*p*=0.013) of students do not have contact with smokers. Regarding the intensity of campaigns against smoking, 47.7% considered it low, 49.6% considered it normal, and 2.7% considered it excessive. Among freshmen, 50% considered the intensity to be low compared with 37.9% of seniors.

As to alcohol consumption, 83.7% of nursing students at the University of Córdoba drink. Of this percentage, 23.1% drink one day or less often per month, 48.1% drink 2 to 4 days per month, 11% drink 2 to 3 days per week, and 1.5% drink 4 or more days per week. Most students in their junior year consumed alcoholic beverages (93.2%; *p*=0.026), followed by those in their senior year (90.5%) (*p*=0.026). Significant differences were found between the last two years of the nursing course and the first two. 

A total of 97.2% students who drink alcohol report that they consume a greater amount on weekends (including Thursday). As to sex, 75% of men consume alcohol compared with 85.5% of women. Regarding the type of alcoholic drink, 23.9% of students usually consume wine, with 19.3% drinking 1-3 glasses per week. A total of 42.8% students usually consume beer, with 35.6% drinking between 1 and 3 medium-sized bottles per week. Of the sample, 36% regularly consume combined drinks, with 27.3% drinking between 1 and 3 times a week. Table 1 shows the consumption of wine, beer, and combined drinks among students by academic year. 

**Table 1:** Characteristics: alcohol and tobacco consumption by academic year (n=264). Córdoba, ES, España, 2016

Variable	Academic Year				P value*
	Fr. year (n=52)	So. year (n=58)	Jr. year (n=59)	Sr. year (n=95)	
Tobacco consumption					0.590
He/She does not smoke, has never smoked	35 (67.3%)	41 (70.7%)	43 (72.9%)	58 (61.1%)	
He/She does not smoke but had smoked	11 (21.2%)	10 (17.2%)	7 (11.9%)	18 (18.9%)	
He/She smokes, but not daily	3 (5.8%)	3 (5.2%)	7 (11.9%)	9 (9.5%)	
He/She smokes daily	3 (5.8%)	4 (6.9%)	2 (3.4%)	10 (10.5%)	
Number of cigarettes consumed per day:					0.554
Fewer than 10	6 (11.5%)	6 (10.3%)	7 (11.9%)	14 (14.7%)	
Between 11 and 20	-	-	1 (1.7%)	3 (3.2%)	
Between 21 and 30	-	1 (1.7%)	-	-	
Smokers on the environment:					
No smokers on the environment	10 (19.2%)	28 (48.2%)	15 (25.4%)	20 (21%)	0.013
Age of onset of tobacco consumption					0.037
Between 13-15 years	-	(1.7%)	(3.4%)	(10.5%)	
Alcohol consumption					0.026
He/She does not drink alcohol	14 (26.9%)	15 (25.9%)	4 (6.8%)	10 (10.5%)	
Once or less a month	7 (13.5%)	17 (29.3%)	15 (25.4%)	22 (23.2%)	
2-4 times/month	26 (50%)	22 (37.9%)	29 (49.2%)	50 (52.6%)	
2-3 times/week	5 (9.6%)	4 (6.9%)	10 (16.9%)	10 (10.5%)	
4 or more times/week	-	-	1 (1.7%)	3 (3.2%)	
Days of the week of greater alcohol consumption:					0.003
Weekends (including Thursdays)	38 (73.1%)	38 (65.5%)	55 (93.2%)	79 (83.2%)	
Consumption of wine as usual	11 (21.2%)	11 (18.9%)	15 (25.4%)	26 (27.4%)	0.297
Consumption of beer as usual	16 (30.8%)	20 (34.5%)	30 (50.9%)	47 (49.5%)	0.136
Consumption of combined drinks as usual	20 (38.4%)	16 (27.5%)	23 (39%)	36 (37.9%)	0.335
Personal opinion about consumption					0.007
He/She thinks he/she does not drink much	(28.9%)	(39%)	(8.5%)	(30.5%)	
University stage:					0.017
It has not influenced him/her, still does not drink alcohol	17 (32.7%)	17 (29.3%)	9 (15.2%)	22 (23.1%)	
He/she consumes less alcohol	3 (5.8%)	8 (13.8%)	6 (10.2%)	18 (18.9%)	

* P value = Chi-Square Test

Of the students, 49.2% began to consume alcoholic beverages between the ages of 16 and 18, 20.5% began between 13 and 15 years, and 16.7% began at 18. No student started drinking alcohol before the age of 13. The amount of alcohol that students believe they drink was significantly different among junior students, with 8.5% (*p*=0.007) believing that they do not drink much, while in other years, the percentage oscillates between 29 and 39%.

The drinking habits of 24.3% of the students have been modified after they entered university, with some consuming less alcohol (13.3%), not drinking at all (2.7%), and drinking more (8.3%). There are significant differences between the academic years. In the junior year, 15.2% of the students (*p*=0.017) state they have not changed their drinking habits after entering university; they still do not consume alcohol. In other years, the percentage ranges from 23 to 33%. There are also significant differences between freshmen and seniors. In the freshman year, 5.8% of students consume less alcohol compared with the 18.9% of those in their senior year (Table 1).

Among the participants, 68.6% of nursing students exercised (Table 2). Women were more physically inactive (35.2%) than men (11.4%). Most university students (52.2%) performed resistance activities, and 32.8% performed several types of physical activities (Table 2). Of the 68.6% students who exercised, 35.6% did it between 3 and 5 days a week, and 23.1% did it twice a week.

**Table 2:** Characteristics: physical activity and diet by academic year (n=264). Córdoba, ES, Spain, 2016

Variable	Academic Year				P value*
	Fr. (n=52)	So. (n=58)	Jr. (n=59)	Sr. (n=95)	
He/she exercises	33 (63.5%)	37 (63.8%)	45 (76.3%)	66 (69.5%)	0.407
University stage:					0.002
He/she exercises less	20 (38.5%)	12 (20.7%)	7 (11.9%)	14 (14.7%)	
He/she exercises more	7 (13.5%)	22 (38%)	22 (37.2%)	33 (34.7%)	
Diet quality:					0.418
Healthy diet	10 (19.2%)	15 (25.9%)	19 (32.2%)	23 (24.2%)	
It needs changes	39 (75%)	41 (70.7%)	40 (67.8%)	66 (69.5%)	
Inadequate	3 (5.8%)	2 (3.4%)	-	6 (6.3%)	

* P value = Chi-Square Test

About entering university, 48.1% of students said their exercise habits had not changed and that they continued performing the same physical activity, while 31.8% reported exercising more. Significant differences were observed in the freshman year, as 38.5% of students (*p*=0.002) reported exercising less. Freshman students (13.5%; *p*=0.002) said they exercised more, while in the other years, the results were between 34 and 38%, with a particular difference among seniors (38.5%). Table 2 shows these results.

A total of 70.5% nursing students needed changes in their diet, 25.4% of students had a healthy diet, and 4.2% had an inadequate diet (Table 2). As to sex, 36.4% of men and 23.2% of women had a healthy diet and 59.1% of men and 72.7% of women needed changes in their diet.

## Discussion

According to the data, 83.7% of nursing students at the University of Córdoba (Spain) consume alcoholic beverages. These results are similar to previous studies on Spanish university students^(^ 11 ^,^ 15 ^-^ 18 ^)^. Alcohol consumption among nursing students in this study is higher than that in other studies with nursing students^(^ 19 ^-^ 20 ^)^ and international studies with university students from Canada^(^ 21 ^)^, Chile^(^ 22 ^)^, Hungary, Lithuania, Slovakia^(^ 23 ^)^, Nigeria^(^ 24 ^)^, and Europe in general^(^ 25 ^)^. Consumption is slightly higher when compared with young people aged between 14 and 18 years^(^ 2 ^)^.

Among the students who drink alcohol, 82.2% consume it fewer than 4 days a week. Students of Córdoba have a weekly consumption similar to those of other studies^(^ 11 ^)^. The most commonly consumed beverage is beer, as in other studies^(^ 26 ^-^ 29 ^)^. This could be due to its low cost and easy access^(^ 26 ^)^.

Seniors drink more alcohol than freshman students, which was found in other studies^(^ 22 ^,^ 30 ^)^. Therefore, being a nursing^(^ 31 ^-^ 32 ^)^ or health sciences^(^ 15 ^)^ student is not a protective factor against alcohol consumption, since there are also more consumers. Most students (97.2%) drink more alcohol on weekends, including Thursday, as other studies also indicate^(^ 11 ^,^ 33 ^-^ 34 ^)^. This may be due to a binge drinking pattern, which lasts few hours and happens on weekends^(^ 2 ^,^ 35 ^)^. The percentage of women who consume alcohol is higher than that of men, which does not coincide with other studies^(^ 15 ^)^. This may be due to the greater episodic alcohol consumption among women^(^ 15 ^)^. 

Of the university students who do not consume alcohol, 60.5% exercise. The data indicate that physical activity could be considered a protective factor against alcohol consumption, as mentioned in other studies^(^ 10 ^,^ 36 ^)^. Nursing students at Córdoba show lower levels of tobacco consumption compared with the general population of 15 to 64-year-olds and with young people aged between 14 and 18 years^(^ 2 ^)^. The percentage of smokers in this study (15.5%) is lower than those obtained in general students by the University of Valencia (24.83%)^(^ 37 ^)^, University of Barcelona (23.4%)^(^ 38 ^)^, University of Vigo (31.7%)^(^ 15 ^)^, University of Leon (29.3%)^(^ 39 ^)^, and University of Texas (26.2%)^(^ 40 ^)^. It is also lower than those obtained in nursing students from the University of Lima (Peru) (17%)^(^ 41 ^)^, Australia (21%)^(^ 42 ^)^, Turkey (22.7%)^(^ 43 ^)^ and (25.2%)^(^ 44 ^)^, Barcelona (35.1%)^(^ 38 ^)^, and University of Chile (23.7%)^(^ 22 ^)^.

As in other studies, the tobacco consumption increased after students entered the university^(^ 11 ^,^ 30 ^,^ 45 ^-^ 46 ^)^. Seniors smoked more than freshmen. There were more smokers in the senior year (14.2% more than in the freshman year), which also occurred with alcohol. Thus, being a nursing^(^ 31 ^-^ 32 ^)^ or health sciences^(^ 15 ^)^ student is not a protective factor against tobacco consumption.

The higher the schooling level, the lower the age of onset of smoking (*p* = 0.037). This early onset of smoking could lead to the use of other types of drugs, such as alcohol and illegal drugs^(^ 40 ^)^. As other studies indicate^(^ 47 ^-^ 51 ^)^, one of the protective factors against smoking is physical activity. This is in agreement with our study, in which 83.9% of students who exercise do not smoke currently. 

Of the students included in this study, 72.3% spent most of the day with a person who smokes occasionally, although only 15.5% of all students smoked. Therefore, having contact with smokers is not a risk factor, as other studies claim^(^ 40 ^,^ 47 ^,^ 52 ^)^. Men smoke more than women. These data coincide with that of other studies^(^ 15 ^,^ 38 ^)^ but disagrees with others^(^ 40 ^,^ 53 ^)^. These differences between studies may be due to the different reasons why people smoke. Women smoke mainly because they believe it helps them stay thin and makes them feel good^(^ 40 ^)^, while men smoke for competition and social relationships^(^ 40 ^,^ 53 ^)^.

In this study, 68.6% of nursing students exercised compared with 31.4% who did not. The number of students that exercise is higher than in other studies^(^ 11 ^,^ 54 ^-^ 55 ^)^ and is similar to the result obtained in studies carried out by the University of United Arab Emirates^(^ 56 ^)^ and University of the Basque Country^(^ 57 ^)^. As to the type of physical activity, more than a half of students practiced resistance activities (52.2%), followed by those who practice two or more types of physical activity (32.8%). 

Of the students in this study, 31.8% said they exercised more after entering university. This situation can also be seen according to the academic year, since 34.7% of students in their senior year said they exercised more compared with 13.5% of those in their freshman year (*p*=0.002). These results indicate that the students of Córdoba exercise more after entering the university. This may be due to individual motivations, such as competition, personal capacity, health improvement, being with friends, and interacting with others^(^ 58 ^-^ 59 ^)^. These motivations can appear at the university stage.

Men exercise more than women, as observed in other studies as well^(^ 15 ^,^ 53 ^-^ 54 ^,^ 60 ^)^. Women exercise mainly out of concern for health and to improve their physical appearance^(^ 60 ^)^. Furthermore, it is in the first stage of adulthood when they most often leave physical activity^(^ 53 ^)^, which usually concurs with the university stage. 

The students had a better food pattern than those in other studies on nursing students from Spain^(^ 14 ^)^, the general population, and people aged between 16 and 24 years^(^ 12 ^)^. A total of 25.4% nursing students had a healthy diet, and 70.5% needed changes in their diet. In a study conducted by the Albacete Faculty of Nursing^(^ 14 ^)^, no student had a healthy diet, and 91.25% needed changes.

Among the students who had a healthy diet, 74.6% exercise compared with 25.4% that do not. Therefore, it seems that physical activity is related to a healthy food pattern, as found in other studies^(^ 53 ^,^ 61 ^)^. With non-sedentary habits, students seem to have regular meal schedules^(^ 61 ^)^.

In comparison, 83.5% of non-smokers had a healthy diet versus 16.5% of smokers. In this study, smoking had a negative effect on food, as other studies have also reported^(^ 62 ^-^ 63 ^)^. A reduced variety of foods was also emphasized^(^ 62 ^)^. 

One limitation of this study is the difficulty of generalizing the results because the sample is limited to nursing students of the University of Córdoba. As usual, another weakness of this type of study is the self-reported information and the cross-sectional design. It is important to pay attention to the reasons why people smoke and drink alcohol in order to develop personalized interventions and promote healthy living habits. Physical activity could be encouraged at the university by providing motivation and resources. It would also be interesting to assess where students live (e.g., in shared flats or at home with parents) to determine how much it affects the variables studied, especially eating habits. In addition, questionnaires with open questions could have been provided for students to express their opinions. This circumstance could offer different themes related to the study. Other university students could also be polled in addition to nursing or health care students at the University of Córdoba. This would allow a fair comparison and make a clear delineation of whether being a nursing student is a protective factor compared with non-health care students.

## Conclusion

In conclusion, nursing students of the University of Córdoba have a high consumption of alcohol and a low consumption of tobacco when compared with other studies. Beer is the most commonly consumed drink, and most students drink more on weekends (including Thursday). As to nutrition, they have a good diet when compared with other studies. Physical activity is related to healthy dieting, and being a smoker has a negative effect on nutrition. Seniors drink more alcohol, smoke more, and exercise more than freshman students. Moreover, the higher the academic year, the lower the age of onset of tobacco consumption. Regarding sex, women drink more alcohol and men smoke and exercise more. The profile of university students can be established as involving high consumption of alcohol, less tobacco consumption, regular physical activity, and they needed diet changes. Finally, being a nursing student cannot be considered a protective factor against alcohol and tobacco consumption; or having good eating habits and exercising.

This study helps us understand the habits of alcohol and tobacco consumption, the practice of physical activity and eating habits in nursing students, who during their studies receive training in healthy habits. As future nursing professionals, they put their health knowledge into practice, since in the future they will have to give advice on healthy habits, and, on the other hand, they will treat patients with addiction to tobacco or alcohol. It would be interesting to reinforce this knowledge and spread it, from the academic period, seminars or courses so that the nursing students promote healthy habits not among themselves but also among other university students in other branches of knowledge or general population.
